# Turning G protein-coupled receptors into tunable biosensors

**DOI:** 10.1093/synbio/ysz011

**Published:** 2019-05-21

**Authors:** Konstantinos Vavitsas

**Affiliations:** Enzyme and Microbial Biotechnology Unit, Department of Biology, National and Kapodistrian University of Athens, Panepistimioupolis, Athens 15784, Greece

In 2012, the Nobel prize in Chemistry was awarded to Robert Lefkowitz and Brian Kobilka for their work in understanding the structure and function of G protein-coupled receptors (GPCRs), one of the largest families of signaling proteins. GPCRs are notoriously difficult to work with (due to their many transmembrane domains) and they operate in a complex way, interacting with several compounds and many internal metabolic pathways. What is more, GPCRs are present in most lifeforms and have an important role in many vital signaling processes. They therefore seem like a very unlikely choice for a biosensor. However, William Shaw and his colleagues proved otherwise in their recent article published in *Cell* (1).

The research group from Imperial College, UK, heavily modified the yeast *Saccharomyces cerevisiae* to obtain a platform for their biosensors. A sensor can be roughly divided into three parts: the detector, the signal transduction/translation component and the reporter. The idea was to create a modular system where GPCRs act as plug-ins dedicated to a compound, while the signal transduction and reporting mechanism will remain the same. *S. cerevisiae* already contains a signaling pathway with its own self-regulation, the MAP kinase cascade, that can be used to translate a GPCR signal to a linear and graded translational response. Using CRISPR-mediated editing the researchers modified 18 genetic loci and removed the rest of the GPCR-related genes, generating a ‘clean’ environment without cross-pathway interactions. In theory, the derived strain can heterologously express a GPCR that recognizes any compound, and receptor activation will stimulate the same pathway and the same reporting event.

Shaw and colleagues modified components of the GPCR receptor and measured the impact on its biosensor properties: the detection threshold, the saturation point and the linearity of response. The experiments took place using the yeast mating pheromone response pathway, where the presence of α-Factor pheromone stimulates a transcriptional response (2). By varying the expression levels of the GPCR components, using different promoters, the researchers showed that it is possible to titrate the signal response, generate a mathematical model with robust predictions and tune the receptor and reporter to function in a certain operational range.

To alter the linearity of response—whether the sensor operates in a linear manner or as an on-off switch—the researchers employed microbial consortia with differently tuned strains. This was displayed in two different scenarios. In the first instance, the presence of melatonin in the media was quantified. The researchers employed two strains with different sensitivities to melatonin, thus increasing the operational range. In the second instance, the presence of the pathogenic fungus *Paracoccidioides brasiliensis* was detected in a yes/no manner. The two cell types used here had different functions: one detected the fungus and released α-Factor pheromone, and the other picked up the pherormone and amplified the signal.

This study displays the potential of using engineered cells as a solution to complex biosensing limitations. This work, an academic collaboration with AstraZeneca, will certainly set the stage for further developing the interplay of synthetic biology and biosensing.
